# A Rare Case of Mucopolysaccharidosis Presenting With Dysostosis Multiplex and Preserved Intelligence in a Seven-Year-Old Girl From Northeast India

**DOI:** 10.7759/cureus.108864

**Published:** 2026-05-14

**Authors:** Pooja Roy, Parvati Roy

**Affiliations:** 1 Pediatrics, North Bengal Medical College and Hospital, Siliguri, IND; 2 Community and Family Medicine, All India Institute of Medical Sciences, Patna, IND

**Keywords:** dysostosis multiplex, glycosaminoglycans, maroteaux-lamy syndrome, mucopolysaccharidosis, scheie syndrome

## Abstract

Mucopolysaccharidoses (MPS) are a group of inherited lysosomal storage disorders caused by a deficiency of enzymes required for the degradation of glycosaminoglycans (GAGs). These disorders are characterized by progressive multisystem involvement, including skeletal deformities, organomegaly, and variable neurocognitive impairment.

We report a case of a seven-year-old girl presenting with recurrent respiratory complaints. Clinical examination revealed coarse facial features, short stature, short neck, hepatomegaly, joint stiffness, corneal clouding, and hearing impairment. Radiological findings showed features of dysostosis multiplex, including J-shaped sella turcica and oar-shaped ribs. Urinary GAG was positive. Cognitive function was normal. Based on clinical and investigative findings, a diagnosis of MPS type I-S (Scheie syndrome) or mild MPS type VI (Maroteaux-Lamy syndrome) was considered. This case highlights the importance of early clinical suspicion of MPS in children presenting with multisystem involvement and skeletal abnormalities, even in the presence of preserved intelligence. Early diagnosis is crucial for timely management and genetic counseling.

## Introduction

Mucopolysaccharidoses (MPS) are a group of inherited metabolic disorders resulting from deficiencies of lysosomal enzymes responsible for the degradation of glycosaminoglycans (GAGs), leading to their accumulation in various tissues [1]. These disorders are mostly inherited in an autosomal recessive manner, except MPS type II (Hunter syndrome), which is X-linked [2].

The clinical spectrum of MPS varies widely, ranging from severe forms with early neurocognitive decline to attenuated forms with preserved intelligence. Common clinical features include coarse facial features, hepatosplenomegaly, skeletal abnormalities (dysostosis multiplex), joint stiffness, cardiac involvement, and corneal clouding [3].

MPS type I (Hurler, Hurler-Scheie, and Scheie syndromes) results from a deficiency of alpha-L-iduronidase, whereas MPS type VI (Maroteaux-Lamy syndrome) is caused by arylsulfatase B deficiency [4]. Attenuated forms such as Scheie syndrome may present later in childhood with milder features and normal intelligence, making diagnosis challenging [5]. Radiological features, particularly dysostosis multiplex, are key diagnostic clues. Urinary GAG analysis serves as a screening tool, while enzyme assay and molecular testing confirm the diagnosis [6].

## Case presentation

A seven-year-old girl presented to the outpatient department with complaints of recurrent cough and cold. She was born at term via normal vaginal delivery with an uneventful perinatal period. She was the product of a non-consanguineous marriage and had an uneventful early developmental history. There was no significant family history.

**Figure 1:**
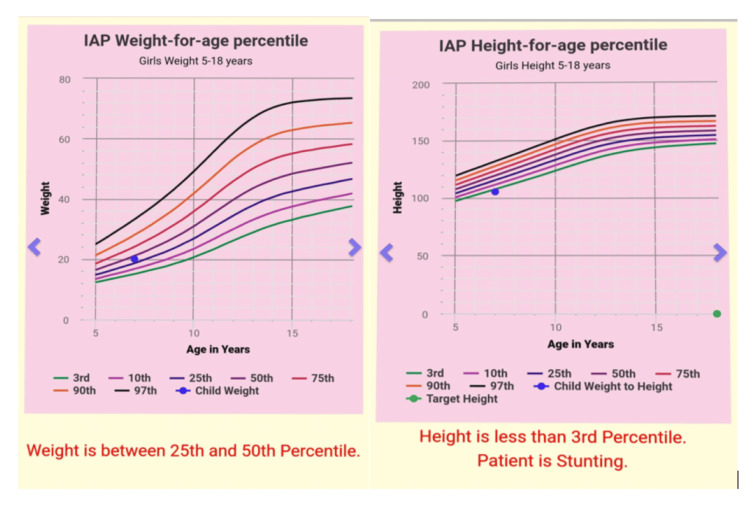
Anthropometric findings of the child showing normal weight-for-age and stunting based on height-for-age. Adapted from the 2015 revised IAP Growth Charts [7]. IAP: Indian Academy of Pediatrics

On examination, the child was alert, conscious, and cooperative. She had coarse facial features, a short neck, and short stature. Anthropometric measurements revealed a weight of 20 kg and a height of 105 cm. The upper segment to lower segment ratio was 0.8:1, and the arm span was 100 cm (Figure 1).

**Figure 2:**
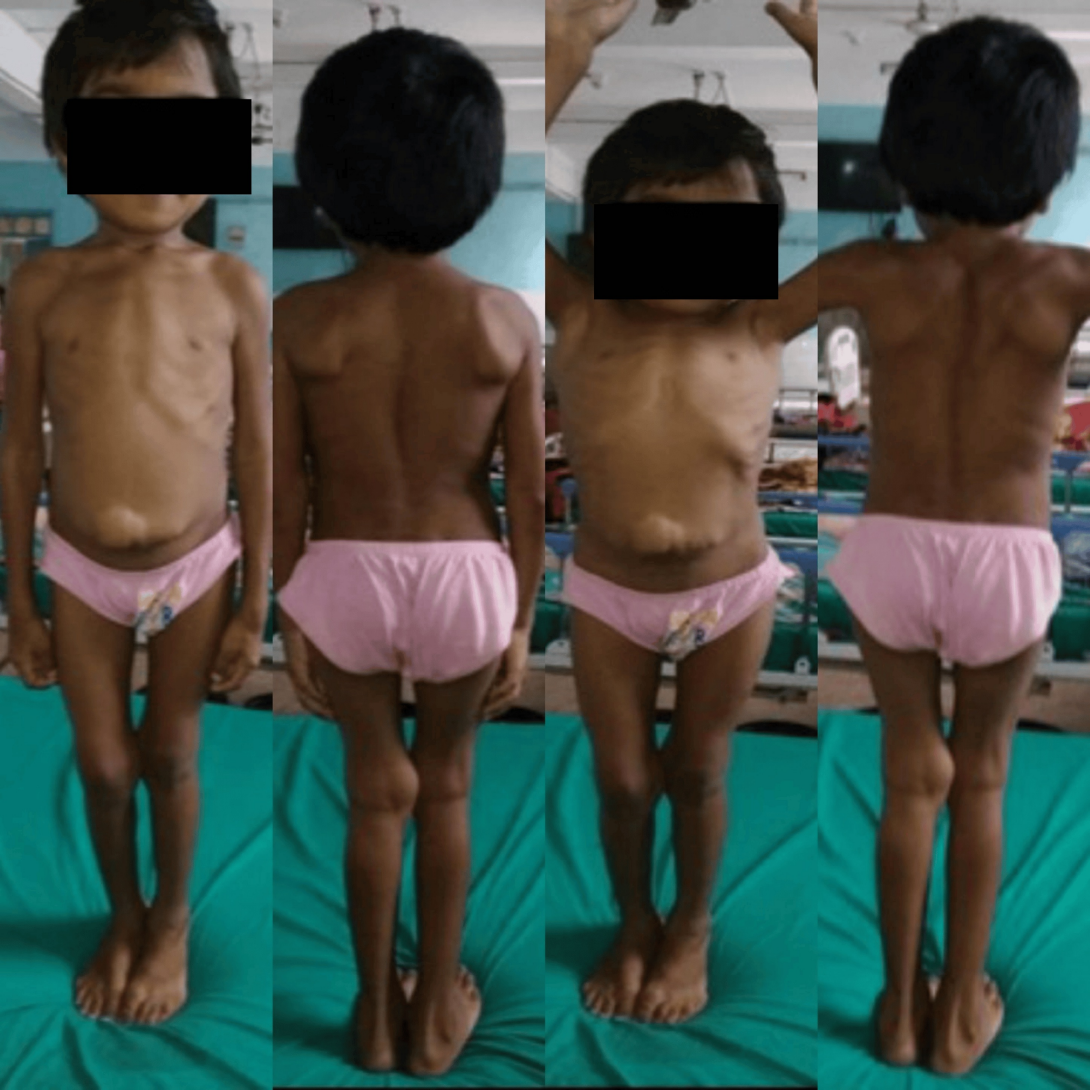
Systemic examination findings of the child showing joint stiffness at elbows and knees and surgical scars from previous umbilical hernia repairs.

Systemic examination showed hepatomegaly and bilateral air entry in the chest. Cardiovascular examination was unremarkable clinically. Additional findings included as follows: hoarse voice, bilateral corneal clouding, conductive hearing loss with otitis media, joint stiffness at elbows and knees, and surgical scars from previous umbilical hernia repairs (Figure 2). Investigation results are shown in Table 1.

**Table 1:** Investigations done in this child revealed elevated urinary glycosaminoglycans (GAG) levels.

Parameters	Observed value	Reference value
Serum calcium	9 mg/dL	8.8-10.8 mg/dL
Serum phosphate	4.5 mg/dL	4-6 mg/dL
Parathyroid hormone	48 pg/mL	10-65 pg/mL
Thyroid-stimulating hormone (TSH)	3.1 mIU/L	0.5-4.5 mIU/L
Free thyroxine (FT4)	1.2 ng/dL	0.9-1.7 ng/dL
Urinary glycosaminoglycans (GAG)	42 mg/mmol creatinine	<10 mg/mmol creatinine
Intelligence quotient (IQ)	91	90-109 (Wechsler Intelligence Scale for Children)

**Figure 3:**
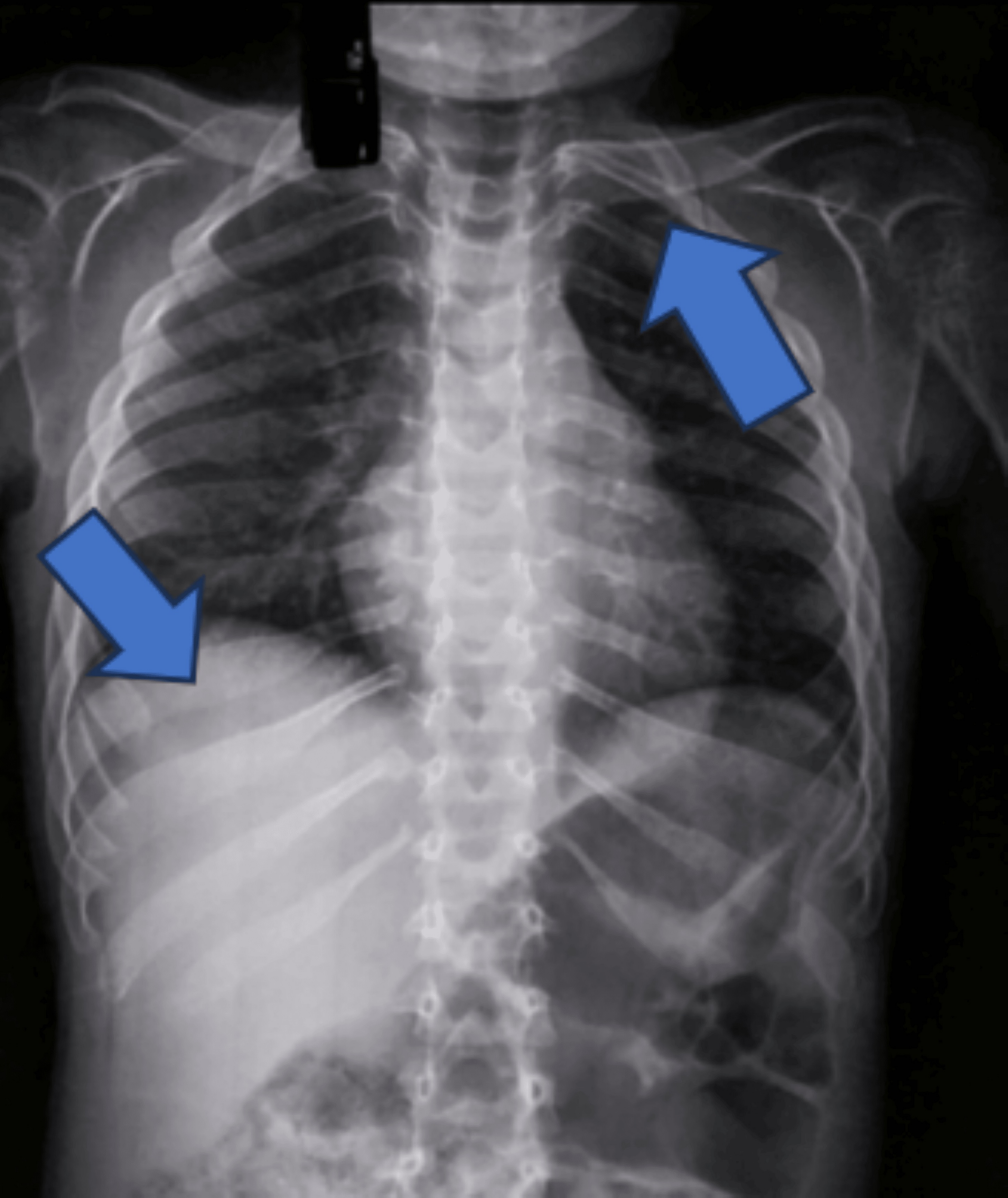
Chest X-ray findings (arrows) of the child showing oar-shaped ribs (9th to 12th ribs) and thickened clavicle.

**Figure 4:**
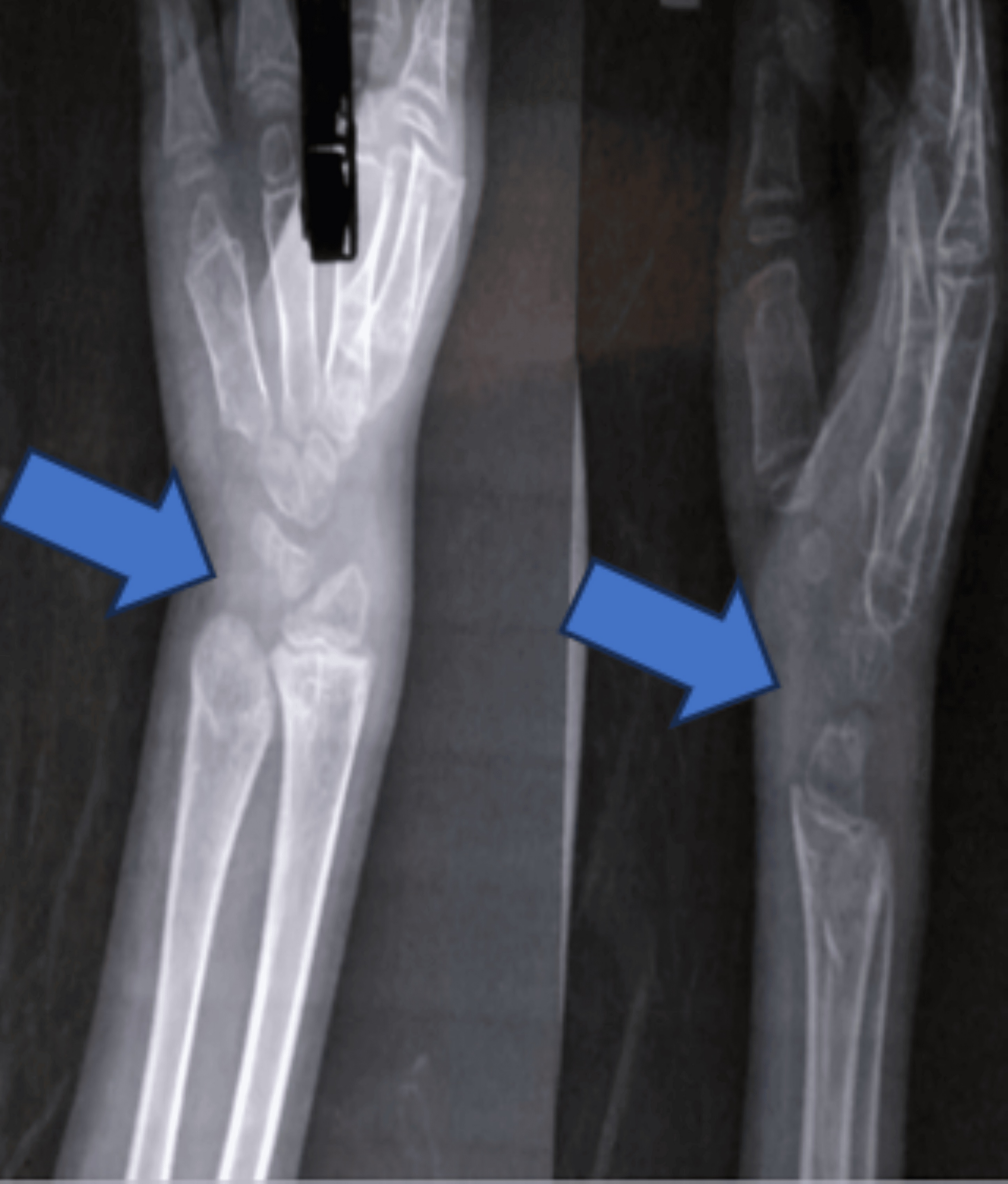
Wrist and hand X-ray findings (arrows) of the child showing features of dysostosis multiplex.

Chest X-ray revealed oar-shaped ribs and a thickened clavicle (Figure 3). Skull X-ray revealed J-shaped sella turcica. Wrist and hand X-ray showed features of dysostosis multiplex (Figure 4). Echocardiography revealed right ventricular enlargement with mild tricuspid regurgitation, and audiometry revealed bilateral conductive hearing loss. An enzyme assay could not be performed due to a lack of facilities.

Based on clinical, radiological, and biochemical findings, a diagnosis of attenuated MPS - likely MPS type I-S (Scheie syndrome) or MPS type VI (Maroteaux-Lamy syndrome) - was made. The patient was referred for higher-center evaluation and counseling regarding enzyme replacement therapy and hematopoietic stem cell transplantation. Symptomatic management focused on respiratory care, hearing evaluation, cardiac monitoring, and orthopedic follow-up.

## Discussion

Mucopolysaccharidoses (MPS) are a heterogeneous group of lysosomal storage disorders characterized by progressive accumulation of glycosaminoglycans (GAGs) due to specific enzyme deficiencies, resulting in multisystem involvement [1,8]. The present case illustrates an attenuated phenotype with classical somatic features but preserved intelligence, which can delay diagnosis and lead to under-recognition in clinical practice [5].

Skeletal abnormalities, collectively termed dysostosis multiplex, are a hallmark of MPS and include features such as J-shaped sella turcica, oar-shaped ribs, and thickened long bones [3]. These findings were evident in our patient and provided an important diagnostic clue. Such radiological features often precede definitive biochemical confirmation and should prompt further evaluation for storage disorders [6].

The presence of corneal clouding and conductive hearing loss in this case supports the likelihood of MPS type I or VI, as these features are commonly observed in both conditions but are typically absent in MPS type II (Hunter syndrome) [4]. Additionally, the preservation of cognitive function favors an attenuated form such as MPS type I-S (Scheie syndrome) or MPS type VI (Maroteaux-Lamy syndrome), both of which may present later in childhood with milder neurological involvement [2,5].

The differential diagnosis in such cases includes conditions like hypothyroidism, rickets, mucolipidosis, and oligosaccharidoses, which may share overlapping features such as growth retardation and skeletal deformities. However, normal thyroid profile and biochemical parameters, along with positive urinary GAG excretion, helped narrow the diagnosis in our case [6].

Furthermore, the burden of disease is significant due to chronic progression, multisystem complications, and impact on quality of life, necessitating early identification and intervention [9]. Epidemiologically, MPS disorders are rare, with variable incidence across populations, contributing to delays in diagnosis, especially in resource-limited settings [10].

Although enzyme assay remains the gold standard for definitive diagnosis, its unavailability in many settings poses a major limitation. In such scenarios, a combination of clinical suspicion, radiological findings, and urinary GAG screening plays a crucial role in diagnosis [6,11]. Early diagnosis is essential, as disease-specific therapies such as enzyme replacement therapy and hematopoietic stem cell transplantation can alter disease progression and improve outcomes [8].

This case highlights the importance of a high index of suspicion for MPS in children presenting with characteristic phenotypic features and skeletal abnormalities, even in the absence of cognitive impairment. Strengthening diagnostic facilities and increasing awareness among clinicians can facilitate early diagnosis, appropriate management, and genetic counseling.

## Conclusions

This case highlights the diagnostic challenge posed by attenuated forms of mucopolysaccharidosis presenting with preserved cognition despite classical multisystem involvement and dysostosis multiplex. The coexistence of coarse facial features, corneal clouding, hearing impairment, hepatomegaly, joint stiffness, and characteristic radiological abnormalities strongly supported a presumptive diagnosis of attenuated MPS. Although definitive enzyme assay and genetic confirmation could not be performed, clinicoradiological correlation, together with elevated urinary glycosaminoglycans, provided substantial supportive evidence. This report emphasizes the importance of maintaining clinical suspicion for MPS even in cognitively preserved children and underscores the practical value of syndromic diagnosis in resource-limited settings where advanced diagnostic facilities may not be readily available. Early recognition enables timely referral, multidisciplinary care, therapeutic planning, and genetic counseling, which may improve long-term outcomes and quality of life.
